# Pulmonary delivery of resveratrol-*β*-cyclodextrin inclusion complexes for the prevention of zinc chloride smoke-induced acute lung injury

**DOI:** 10.1080/10717544.2022.2048135

**Published:** 2022-04-05

**Authors:** Wanmei Wang, Yan Liu, Pan Pan, Yueqi Huang, Ting Chen, Tianyu Yuan, Yulong Ma, Guang Han, Jiahuan Li, Yiguang Jin, Fei Xie

**Affiliations:** a Pharmaceutical College of Henan University, Kaifeng, China; b Department of Pharmaceutical Sciences, Beijing Institute of Radiation Medicine, Beijing, China; c Respiratory Intensive Care Unit, The First Medical Center of Chinese PLA General Hospital, Beijing, China; d Department of Anesthesiology, The First Medical Center of Chinese PLA General Hospital, Beijing, China

**Keywords:** Pulmonary delivery, zinc chloride smoke, resveratrol, *β*-cyclodextrin, acute lung injury

## Abstract

Smoke bombs are often used in military/fire training, which can produce a large amount of zinc chloride (ZnCl_2_) smoke. Inhalation of ZnCl_2_ smoke usually causes acute lung injury (ALI) that would likely develop to acute respiratory distress syndrome (ARDS). However, there is no effective prevention or treatment strategy for the smoke-induced ALI. Resveratrol (RES) is a natural polyphenol with good anti-inflammatory and anti-apoptotic activities, but its low solubility, stability, and bioavailability restrict its clinical application. In this study, an inhalable RES formulation composed of RES-*β*-cyclodextrin inclusion complexes (RES-*β*-CD) was prepared for the prevention of ZnCl_2_ smoke-induced ALI. RES-*β*-CD powders had a small mass median aerodynamic diameter of 3.61 μm and a high fine particle fraction of 38.84%, suitable for pulmonary inhalation. RES-*β*-CD exhibited low BEAS-2B cytotoxicity. Pulmonary delivery of RES-*β*-CD to mice remarkably prevented the smoke-induced ALI with downregulation of TNF-*α*, IL-1*β*, STAT3, and GATA3, and upregulation of T-bet and Foxp3. RES-*β*-CD protected the respiratory function, percutaneous oxygen saturation, physical activity, lung capillary integrity, and lung liquid balance, alleviating inflammation and apoptosis. Pulmonary delivery of the positive drug, budesonide (BUD), also alleviated the smoke-induced ALI by reduction of inflammation and cell apoptosis. RES-*β*-CD exhibited the regulation of the Th1/Th2 and Treg/Th17 balances, while BUD did not show any effect on immune balances. In conclusion, pulmonary delivery of RES-*β*-CD is a promising anti-inflammatory and anti-apoptosis strategy for the prevention of ZnCl_2_ smoke-induced ALI by direct lung drug distribution and regulation of immune balance.

## Introduction

Smoke bombs produce a large amount of barrier smoke as soon as ignition, which are widely used for shielding in the withdraw or attack of troops or anti-terror policemen, battlefield simulation in military/fire drills, and firefighter training (Gil et al., 2008; Xie et al., 2019). The produced smoke (ZnCl_2_ smoke) mainly contains zinc chloride (ZnCl_2_), zinc oxide, and other chemical ingredients. Inhalation of the high doses of ZnCl_2_ smoke would likely cause acute lung injury (ALI), even then develop acute respiratory distress syndrome (ARDS) (Mahboob et al., 2017).

Although the ZnCl_2_ smoke-induced ALI occasionally happens, its mechanism is still unclear. Common therapies including corticosteroids, antibiotics, and supplemental oxygen or positive pressure ventilation may be used but these treatments do not show definite efficiency for the ALI induced by ZnCl_2_ smoke. An effective prevention and control method is urgent for the ALI induced by ZnCl_2_ smoke.

Resveratrol (RES) is a natural polyphenol compound, abundantly present in many common fruits and vegetables, and used as healthy food (Zhao et al., 2020). RES owns a variety of biological activities, such as anti-inflammation (Lu et al., 2019), anti-apoptosis (Song et al., 2017), anti-oxidation (Abdu & Al-Bogami, 2019), and antitumor effect (Zhao et al., 2020). Furthermore, RES is reported as a treatment of ALI/ARDS by inhibition of inflammatory response, prevention of cell apoptosis, and anti-oxidation (Jiang et al., 2016; Zhu et al., 2020). Inhaled RES inhibited the nuclear translocation of nuclear factor-κB and decreased the expression of pro-inflammatory cytokines for the treatment of lipopolysaccharide-induced ALI (Kim et al., 2018). Unfortunately, RES has been not clinically applied, though some clinical trials involving RES-contained formulations have been conducted for treatment of diabetes, obesity, cancer, neurological and cardiovascular diseases (Singh et al., 2019). The major reasons that prevent RES from clinical applications include its poor water solubility, weak stability, and low bioavailability (Huang et al., 2019).

*β*-Cyclodextrin (*β*-CD) is a macrocycle compound composed of seven *α*-d-glucopyranoside units. Hydrophobic drugs of proper molecular volume can entry into the cylinder caves of *β*-CD to form inclusion complexes, improving drug solubility, stability, and bioavailability, and reducing toxicity (Liao et al., 2016). Furthermore, *β*-CD can improve drug absorption after pulmonary delivery (Mohtar et al., 2017).

Pulmonary drug delivery is a noninvasive method to deliver drugs to damaged lung tissues or improve systemic absorption from the lung (Walenga & Longest, 2016). The ideal therapeutic way of lung diseases is pulmonary drug delivery, involving asthma (Chandel et al., 2019), chronic obstructive pulmonary disease (Wallin et al., 2018), pneumonia (Li et al., 2017), ALI (Zhang et al., 2021), and pulmonary fibrosis (Hu et al., 2018). Currently, the major types of pulmonary delivery strategies include pressurized metered-dose inhalers, nebulizers, dry powder inhalers (DPIs), and soft mist inhalers (Peng et al., 2016). DPIs are composed of portable solid powders. The advantages of DPIs include high drug loads, portability, propellant-free, and good stability (Zhang et al., 2020). However, the development of DPI formulations is a challenge for many drugs because of their high-dose needs and improper physicochemical properties.

Here, we prepared inhalable RES-*β*-cyclodextrin inclusion complexes (RES-*β*-CD) for the prevention of ALI induced by ZnCl_2_ smoke. To our knowledge, this is the first study of inhaled natural products for the prevention of ZnCl_2_ smoke-induced ALI. RES-*β*-CD was intratracheally (i.t.) administered to ZnCl_2_ smoke-induced ALI mouse models. The effects and mechanisms of RES-*β*-CD against ZnCl_2_ smoke-induced ALI were investigated.

## Materials and methods

### Materials

RES was purchased from Shanghai Aladdin Biochemistry Technology Co., Ltd. (Shanghai, China). *β*-CD was purchased from Beijing Innochem Technology Co., Ltd. (Beijing, China). Budesonide Suspension for Inhalation was purchased from AstraZeneca Pty Ltd. (Sydney, Australia). Evans blue was purchased from Acros Organics (Geel, Belgium). All other chemicals and solvents were of analytical grade or high-performance liquid chromatographic (HPLC) grade. Bronchial epithelial cell media (BEpiCM) were purchased from ScienCell Research Laboratories, Inc. (Carlsbad, CA).

### Animals and cells

Male ICR mice (25 ± 2 g) were purchased from the SPF (Beijing) Biotechnology Co., Ltd. (Beijing, China). All experimental procedures were executed according to the protocols approved by the Institutional Animal Care and Use Committee at Beijing Institute of Radiation Medicine. Normal human bronchial epithelial cells (BEAS-2B cells) were provided by Beijing Institute of Radiation Medicine (Beijing, China), which were grown in the BEpiCM media and incubated at 37 °C in the humidified 5% CO_2_ environment.

### Preparation and characterization of RES-*β*-CD

RES-*β*-CD was prepared with the grinding method. Briefly, an RES solution in ethanol was mixed with a *β*-CD aqueous solution (RES:*β*-CD, 1:1, mol/mol) followed by continuous grinding for 1 h. The suspension was freeze-dried for 40 h in a lyophilizer (LGJ-30F, Beijing Songyuan Huaxing Technology Develop Co., Ltd., Beijing, China).

The thermal behavior of RES, *β*-CD, their physical mixture (RES/*β*-CD, 1:1, mol/mol) and RES-*β*-CD was investigated on a differential scanning calorimeter (DSC, Q2000, TA, New Castle, DE). The crystalline states of them were investigated with an X-ray powder diffraction (XRD, Ultima IV, Rigaku, Tokyo, Japan).

The microscale morphologies of RES, *β*-CD, and RES-*β*-CD powders were observed under a scanning electron microscope (SEM, CUBE2, EmCrafts, Gyeonggi-do, Korea). The repose angle of RES-*β*-CD powders was measured using the funnel method. The volume diameter was defined as the geometric mean diameter (*D*_50_) and measured with a particle size analyzer (BT2001, Bettersize Instruments Ltd., Dandong, China) based on the laser light diffraction method.

The simulated lung deposition of RES-*β*-CD was explored on the Next Generation Impactor (NGI, TPK 2000R, Copley, Nottingham, UK) with an inspiratory flow rate of 60 L/min. Approximately 10 mg of RES-*β*-CD powders were filled in hydroxypropyl methylcellulose (HPMC) hard capsules (Capsugel^®^, Type 3, Suzhou Capsule Ltd., Suzhou, China) and placed into a DPI device (Type 006, Shanghai Huarui Aerosol Co., Ltd., Shanghai, China) for the deposition test. Deposition was repeated with 20 capsules. The deposited powders were collected from each stage and separately dissolved in methanol for HPLC measurement of RES. The fine particle fraction (FPF), mass median aerodynamic diameter (MMAD), and geometric standard deviation (GSD) were calculated using the software (Copley Inhaler Testing Date Analysis Software, Version 3.10 Wibu USP 32/Ph. Eur.6.0, Copley, Nottingham, UK).

RES-*β*-CD was dissolved in methanol to extract RES that was measured with HPLC after filtration through 0.22 μm membranes. The HPLC system included an Agilent 1260 system (Santa Clara, CA) equipped with an Agilent C18 column (250 mm × 4.6 mm, 5 µm) at 25 °C, the mobile phase of acetonitrile/water (30:70, v/v) at a flow rate of 1.0 mL/min, the injection volume of 20 µL, and the wavelength of 306 nm. Entrapment efficiency and loading efficiency were calculated with encapsulated RES/total RES × 100% and encapsulated RES/total RES-*β*-CD × 100%, respectively.

### Cytotoxicity assay

BEAS-2B cells were seeded in 96-well plates at a density of 2 × 10^4^ cells/well and incubated at 37 °C for 24 h. A series of RES-*β*-CD suspensions in BEpiCM media with RES concentrations ranging from 0.625 µmol/L to 80 µmol/L were added into the wells, respectively, and the cells were incubated for 12 h. The plates were washed once with sterilized phosphate-buffered solutions (PBS). The culture media containing 10% cell counting kit-8 (CCK-8, Beiren Chemical Technology (Beijing) Co., Ltd., Beijing, China) were added into each well followed by incubation for 2.5 h. The absorbance of wells was measured at 450 nm with a microplate reader (ELx800, BioTek Instrument, Winooski, VT) and the cell viability was calculated as (absorbance of sample – absorbance of blank)/(absorbance of control – absorbance of blank)×100%.

### ALI models induced by smoke bombs

Smoke bombs composed of zinc oxide, hexachloroethane, aluminum powder, silicon powder, and black powder were prepared. An aliquot (3 g) of the smoke bomb was put in a transparent cube chamber (1 m × 0.8 m × 0.6 m) with a door. A mouse cage was equally divided into six zones with plates to ensure every mouse having independent space and equal inhalation of smoke. The mouse-loaded cage was placed in the chamber, the smoke bomb was ignited, and the door was closed. Three minutes later, the cage was withdrawn and all the mice were moved to a new clean cage.

### Pharmacodynamic study

The mice were randomly divided into four groups (10 each group). The mice in the model group were i.t. administrated with saline to simulate the administration process. The mice in the BUD group and the RES-*β*-CD group were i.t. administrated with BUD (1 mg/kg) suspensions and RES-*β*-CD (80 mg/kg, containing 12.66 mg RES/kg, approximately 50 μL) suspensions in saline, respectively. The dose of RES-*β*-CD was used according to our preliminary experiments and the published report (Ma et al., 2016; Kim et al., 2018). All the medicines were i.t. sprayed into the mouse lungs using a homemade dosing needle after the mice were anesthetized. ZnCl_2_ smoke exposure began within 30 min after administration to the mice. The untreated mice were as the healthy group. ZnCl_2_ smoke exposure was continued for 3 min. Physiological and pathological tests began 48 h post-smoke exposure, involving respiratory function, movement ability, and percutaneous oxygen saturation (SpO_2_). The lung tissues were excised after sacrifice and their surface liquids were removed with filter paper. The left lungs were weighed to get the wet weight (*W*) of lungs and completely dried at 60 °C for 48 h to get the dry weight (*D*). Lung *W*/*D* ratios were calculated. The upper lobes of the right lungs were immersed in 10% formalin solutions and then embedded in paraffin. The pathological sections were obtained and stained with hematoxylin and eosin (H&E).

### Assessment of physiological functions

An unrestrained whole body plethysmograph (EMKA Technologies, Paris, France) was used for measurement of the peak expiratory flow (PEF), expiratory flow at 50% tidal volume (EF50), and tidal volume (TV) of the mice. Respiratory indexes were automatically recorded for 5 min.

A finger clip pulse oximeter (MD300C, Beijing Chaosi Electronic Technology Co., Ltd., Beijing, China) was clamped at the tail of mice for measurement of SpO_2_.

The physical activity of mice was evaluated with a rotating rod fatigue tester (DB093, Beijing Zhishu Duobao Biological Technology Co., Ltd., Beijing, China). Each mouse was trained at 10 r/min for 20 s before the experiment began. The total test time was set to 180 s. The rotation speed of rod (93 mm in diameter) gradually increased from 0 r/min to 38 r/min within 20 s and maintained to the end. The number of dropping times was recorded.

### Assessment of pulmonary vascular permeability

The mice were randomly divided into four groups (four each group). All the animal experimental processes were the same as the above pharmacodynamic study until 47 h post-smoke exposure. An Evans blue saline solution (2%, 20 mg/kg) was injected to the mice via tail veins for evaluation of pulmonary vascular permeability. One hour post-injection, the mice were anesthetized with chloral hydrate and the peripheral blood was almost removed out by cutting of the abdominal aorta. The heart and lung were together withdrawn. The cardiopulmonary circulation was flushed with saline until no residual blood in the lung. The lung was departed from the heart and photographed. The left lung was homogenized in formamide and incubated at 37 °C for 24 h. The homogenates were centrifuged (5000×*g*, 10 min) in a centrifuge (H2-16 KR, Hunan Kecheng Instrument Equipment Co., Ltd., Changsha, China) and the supernatants were collected. The optical densities of samples were measured at 630 nm. The concentrations of Evans blue in the homogenates were calculated according to the standard curve of Evans blue in formamide.

### Measurement of cytokines

The levels of tumor necrosis factor-alpha (TNF-*α*) and interleukin-1 beta (IL-1*β*) in the homogenated supernatants of the middle and lower lobes of the right lungs were detected with the ELISA kits (Shanghai Enzyme-linked Biotechnology Co., Ltd., Shanghai, China) according to the instructions.

### Apoptosis assay

The above paraffin-embedded upper lobes of the right lungs were used for apoptosis assay. The terminal deoxynucleotidyl transferase biotin-dUTP nick end label (TUNEL, Beijing Solarbio Science & Technology Co., Ltd., Beijing, China) staining was conducted followed by incubation of tissue sections for 60 min at 37 °C. The sections were washed with PBS (pH 7.4), and then incubated with DAPI (4,6-diamidino-2-phenylindole) for 10 min at room temperature for detection of nucleoli. The images of TUNEL and DAPI fluorescence were obtained. DAPI and apoptosis positive cells were enumerated using the ImageJ software (The National Institutes of Health, Bethesda, MD). Apoptosis ratios were calculated as the number of apoptotic cells/the number of DAPI.

### Immunohistochemistry

The right upper lobes of the lungs embedded in paraffin were deparaffinized in xylene and rehydrated with ethanol. The tissues were immersed in ethylenediamine tetraacetic acid (EDTA) antigen retrieval solutions (pH 8.0). The antigens were removed after microwave-heating for 15 min. The samples were washed with water and processed with 3% H_2_O_2_ solutions to remove the endogenous peroxidases. The primary antibody of T-box transcription factor (T-bet), GATA binding protein 3 (GATA3), signal transducer and activator of transcription 3 (STAT3), and forkhead box P3 (Foxp3) diluted with PBS (pH 7.4) was applied and incubated for 30 min at room temperature. The secondary antibody of the primary antibody was applied for 30 min at room temperature with interval PBS washing for immunohistochemical detection. Brown areas indicated positive cells. The average optical density (AOD) of positive cells was calculated as the integral optical density/the area with the ImageJ software (The National Institutes of Health, Bethesda, MD). All the reagents were obtained from Beijing Solarbio Science & Technology Co., Ltd. (Beijing, China).

### Statistical analysis

Data were presented as mean ± standard deviation (SD). All statistical analyses were performed using the SPSS 16 software (SPSS, Chicago, IL). One-way ANOVA test was performed for all the statistical evaluations. Pairwise multiple comparisons between groups were identified using the LSD test. Statistical significance was defined as *p* < .05.

## Results

### Characteristics of RES-β-CD

**Figure 1.**
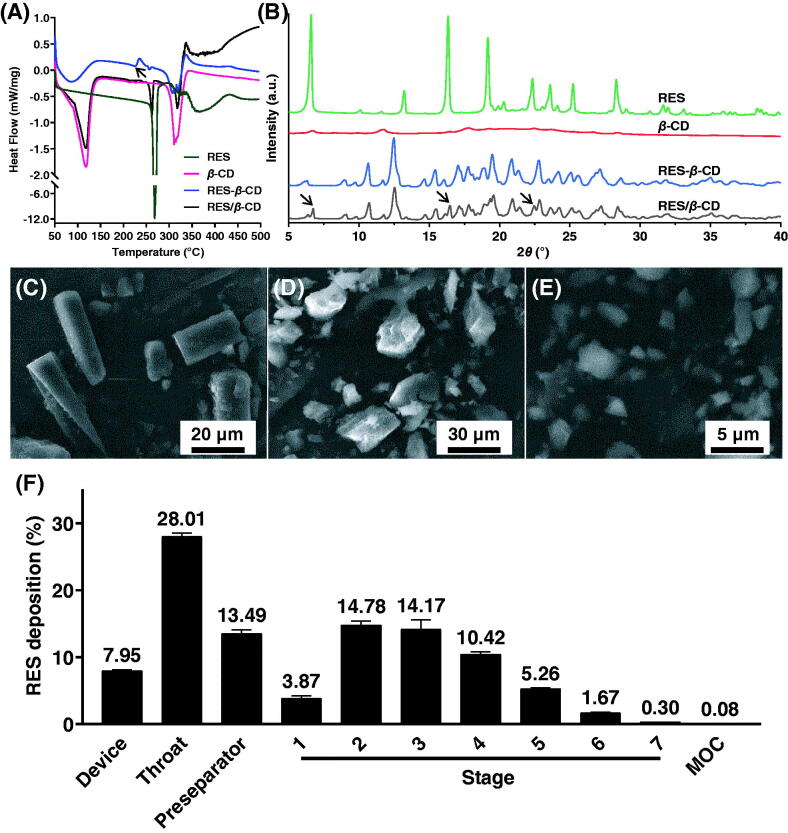
Characteristics and deposition of RES-*β*-CD. DSC graphs (A), XRD graphs (B) of RES, *β*-CD, RES-*β*-CD, and RES/*β*-CD. The arrow in graph (A) indicates a new endothermic peak. The arrows in graph (B) indicate the peaks disappearing in RES-*β*-CD. SEM images of RES powders (C), *β*-CD powders (D), and RES-*β*-CD powders (E). The simulated RES lung deposition of RES-*β*-CD in the NGI (F). Data are presented as mean ± SD (*n* = 3). MOC: micro-orifice collector.

The formation of inclusion complexes can be verified with many physical methods. Here, we used DSC and XRD to identify the formation of RES-*β*-CD. RES-*β*-CD showed a different DSC graph from RES, *β*-CD, and RES/*β*-CD with a new endothermic peak at 224.89 °C while RES/*β*-CD only showed an overlaying graph of those of RES and *β*-CD (Figure 1(A)). Three 2*θ* peaks at 6.72°, 16.46°, and 22.48° disappeared in the XRD graph of RES-*β*-CD compared to those of RES/*β*-CD (Figure 1(B)). Therefore, the formation of RES-*β*-CD was confirmed.

The particles in RES raw materials powders were cylinder crystals, while both *β*-CD particles and RES-*β*-CD particles were irregular according to the SEM images (Figure 1(C–E)). Moreover, the sizes of these particles were of great difference. RES particles and *β*-CD particles were large to 20–40 µm in diameter, while the size of RES-*β*-CD particles was only 4 µm or less. The *D*_50_ of RES-*β*-CD powders was 3.54 ± 0.01 µm (*n* = 3). In addition, the flowability of RES-*β*-CD powders was good with a small repose angle of 32.23 ± 0.64° (*n* = 3), favoring the filling of powders into capsules. The entrapment efficiency and loading efficiency of RES-*β*-CD were 94.91% and 15.82%, respectively.

### Highly efficient lung deposition of RES-β-CD

The lung deposition or pulmonary delivery efficiency of particles is determined by their aerodynamic diameter. The MMAD, FPF, and GSD of RES-*β*-CD powders were 3.61 ± 0.10 µm, 38.84 ± 2.07%, and 1.77 ± 0.01 (*n* = 3), respectively, indicating that RES-*β*-CD powders would have effective lung deposition when inhalation (Figure 1(F)).

### RES-β-CD alleviated ZnCl_2_ smoke-induced ALI

**Figure 2.**
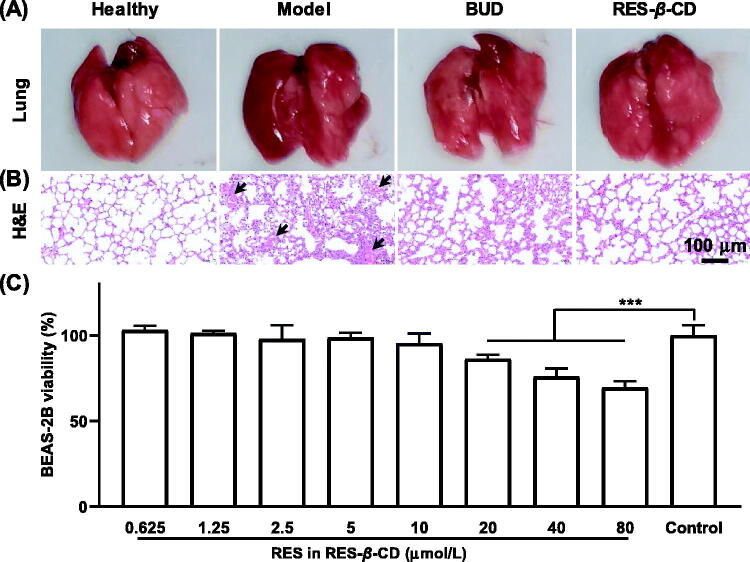
RES-*β*-CD alleviated ZnCl_2_ smoke-induced ALI. Appearance of lung tissues (A). The images of H&E staining (B). The arrows indicate the significant hemorrhage sites of the lungs. BEAS-2B cell viability (C). Data are presented as mean ± SD (*n* = 6). ***p < .001.

ZnCl_2_ smoke-induced ALI was followed by serious syndromes. The mice expressed shortness of breath and the excised lungs were dark-red with the significant edema and a lot of bleeding compared to the smooth and red lungs of the healthy mice (Figure 2(A,B)).

All the mice in the BUD and RES-*β*-CD groups had normal physical activity, and a little hemorrhage and the basically clear alveolar structures in the lungs, indicating remarkable alleviation of the ALI induced by ZnCl_2_ smoke, while the RES-*β*-CD group seemed better than the BUD group (Figure 2(A,B)). By contrast, RES-*β*-CD had no effect on the growth of BEAS-2B cells even up to a high concentration of 10 µmol/L (Figure 2(C)), indicating the safety of RES-*β*-CD at high concentration. Therefore, pulmonary delivery of RES-*β*-CD is a promising strategy for prevention of ZnCl_2_ smoke-induced ALI.

### RES-β-CD alleviated pulmonary edema and vascular leakage

**Figure 3.**
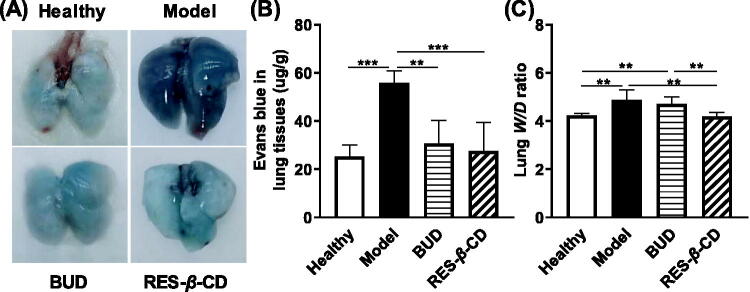
RES-*β*-CD alleviated pulmonary edema and vascular leakage. Appearance of the lungs after Evans blue was intravenous injected 48 h post-ZnCl_2_ smoke exposure (A), the amounts of Evans blue in the lung tissues (B, *n* = 4), and the lung *W*/*D* ratios (C, *n* = 5). Data are presented as mean ± SD. **p < .01 and ***p < .001.

In this study, we detected pulmonary vascular permeability by the extravasation of this dye into the pulmonary extravascular space and the surrounding lung tissues. The lungs of model mice were obviously blue after 48 h post-smoke exposure; however, the mice administrated with BUD and RES-*β*-CD only showed little blue lungs compared to the healthy lung (Figure 3(A)). The content of Evans blue in the lung tissues of model mice was much more than those in the prevented mice and healthy mice while no significant difference was presented between the latter two groups (Figure 3(B)). Moreover, we used the lung *W*/*D* ratios to evaluate the extent of pulmonary edema. ZnCl_2_ smoke exposure markedly increased the lung *W*/*D* ratios while the prevention of RES-*β*-CD and BUD decreased the *W*/*D* ratios. However, the effect of RES-*β*-CD was much higher than that of BUD (*p* < .01) (Figure 3(C)). Therefore, RES-*β*-CD greatly ameliorated pulmonary vascular leakage and pulmonary edema induced by ZnCl_2_ smoke exposure.

### Anti-inflammation and anti-apoptosis effects of RES-β-CD

**Figure 4.**
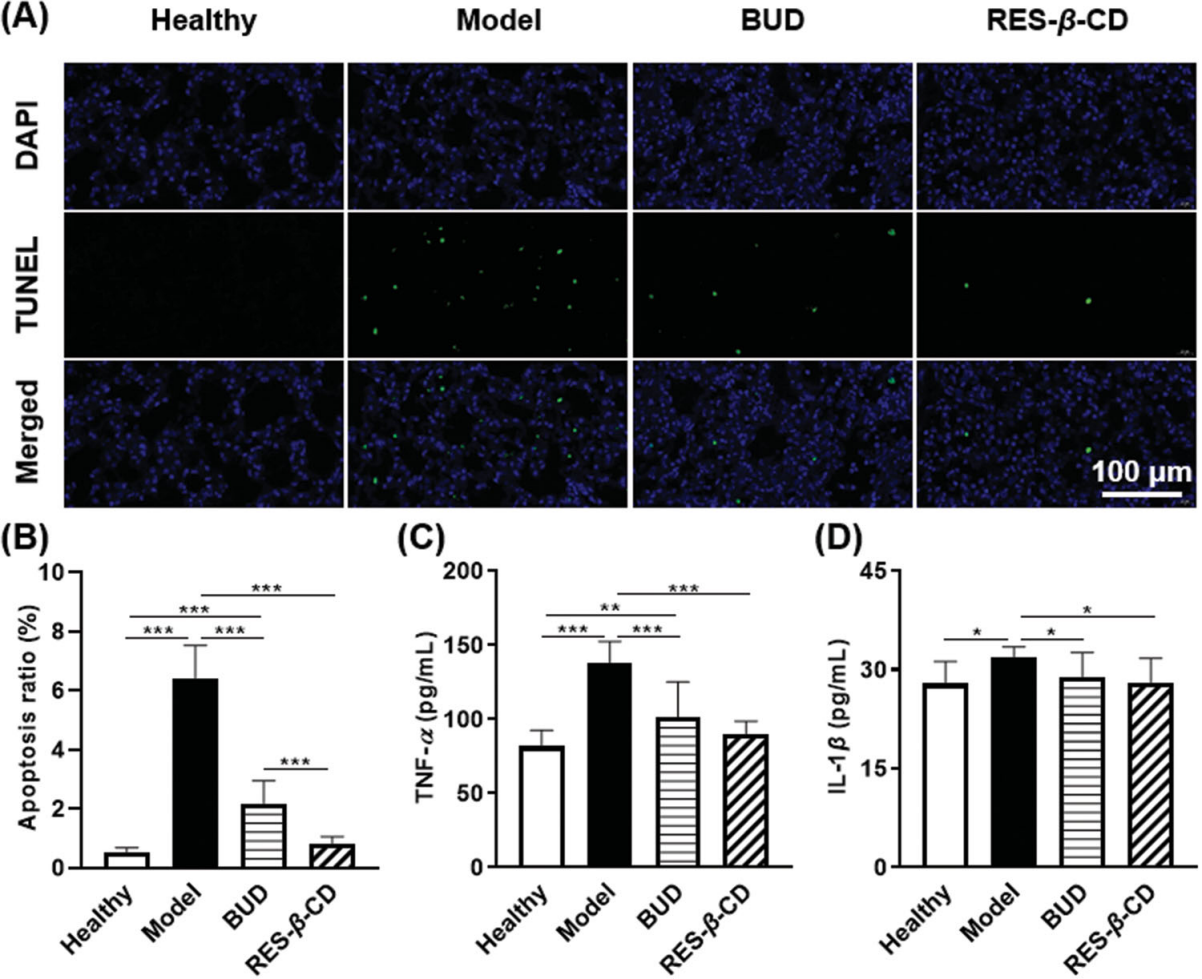
Anti-inflammation and anti-apoptosis effects of RES-*β*-CD. TUNEL and DAPI images of the lung tissues (A). The apoptosis ratio (B, *n* = 15). Expressions of TNF-*α* and IL-1*β* in the lung tissues (C and D, *n* = 10). Data are presented as mean ± SD. *p < .05, **p < .01, and ***p < .001.

Apoptosis of lung tissue cells is usually the result of lung injury (Huang et al., 2020). Here, we demonstrated that ZnCl_2_ smoke exposure observably improved the apoptosis of mouse lung tissue cells with the high TUNEL numbers (Figure 4(A)). Both RES-*β*-CD and BUD remarkably attenuated apoptosis, where the effect of RES-*β*-CD was much stronger than that of BUD (*p* < .001) (Figure 4(A,B)). The high anti-apoptosis effect of RES-*β*-CD is confirmed after ZnCl_2_ smoke exposure and inhalation into the lung.

The expression of two typical pro-inflammatory cytokines in the lung tissues, i.e. TNF-*α* and IL-1*β*, was reduced by BUD and RES-*β*-CD, especially for TNF-*α*, after ZnCl_2_ smoke exposure (Figure 4(C,D)). Therefore, RES-*β*-CD is a good anti-inflammation agent for the ALI induced by ZnCl_2_ smoke.

### RES-β-CD increased the SpO_2_, maintained the respiratory functions and the physical activity of ALI mice

**Figure 5.**
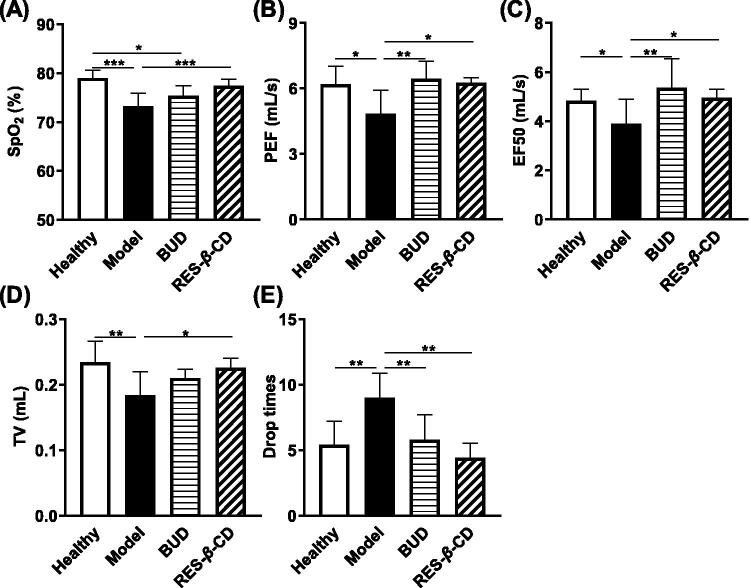
RES-*β*-CD increased the SpO_2_, maintained the respiratory functions and the physical activity of ALI mice. Levels of SpO_2_ (A), PEF (B), EF50 (C), TV (D), and drop times (E). Data are presented as mean ± SD (*n* = 5). *p < .05, **p < .01, and ***p < .001.

ZnCl_2_ smoke exposure obviously reduced the SpO_2_ of mice. Interestingly, RES-*β*-CD increased the SpO_2_ of ZnCl_2_ smoke exposed mice, but BUD did not (Figure 5(A)). RES-*β*-CD could effectively increase SpO_2_ by attenuating the ZnCl_2_ smoke-induced ALI.

ZnCl_2_ smoke exposure reduced the levels of TV, PEF, and EF50 in the mice. However, such changes disappeared in the RES-*β*-CD treated mice (Figure 5(B–D)), so that RES-*β*-CD maintained the respiratory function of ALI mice.

Moreover, the ZnCl_2_ smoke-exposed mice showed much more drop times from the rotating rod fatigue tester than the healthy mice (Figure 5(E)). Both RES-*β*-CD and BUD prevented the dropping of the mice from the tester with the comparable drop times to the healthy mice (Figure 5(E)). This result was consistent with the above results.

### RES-β-CD regulated the balances of Th1/Th2 and Treg/Th17

**Figure 6.**
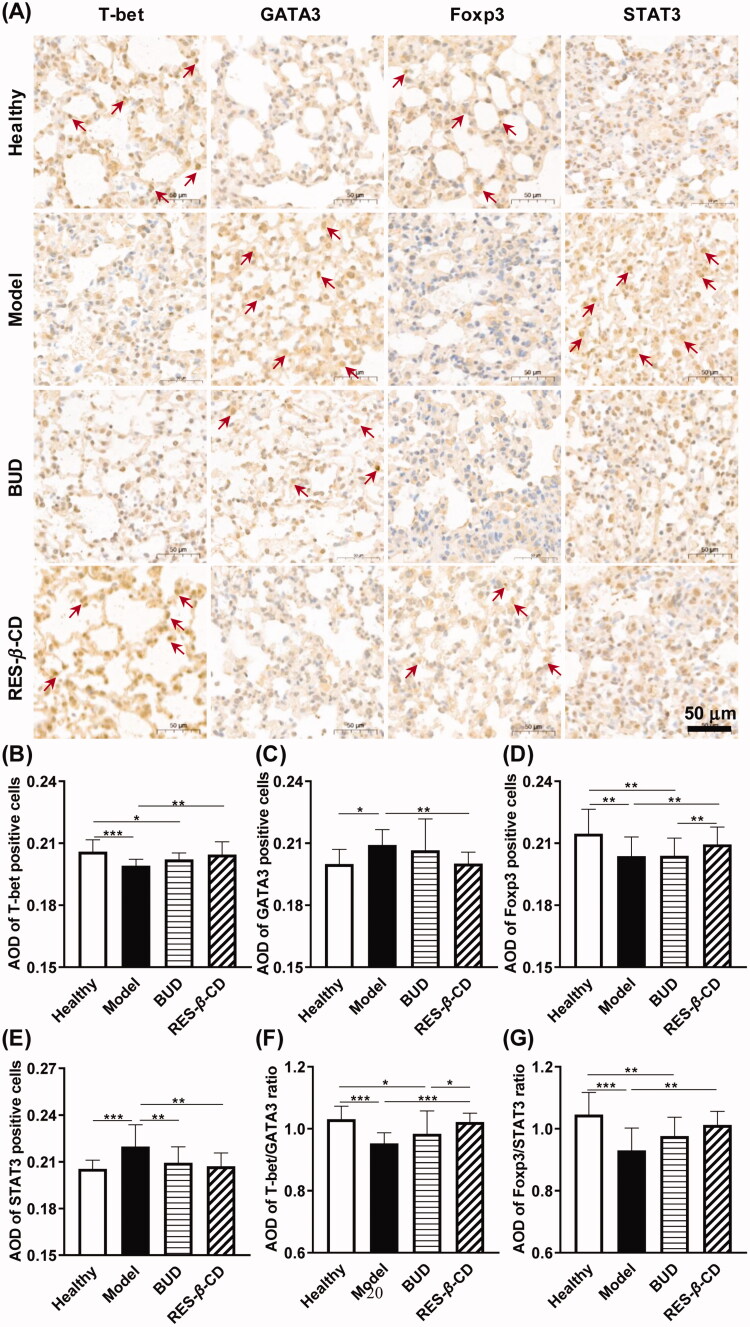
RES-*β*-CD regulated the balances of Th1/Th2 and Treg/Th17. Expressions of T-bet, GATA3, Foxp3, and STAT3 in the lung tissues (A). The arrows indicate the expressions of T-bet, GATA3, Foxp3 and STAT3 that are brown-stained. AOD of T-bet (B), GATA3 (C), Foxp3 (D), and STAT3 (E) positive cells. The ratio of the AOD of T-bet/GATA (F) and Foxp3/STAT3 (G). Data are presented as mean ± SD (*n* = 15). *p < .05, **p < .01, and ***p < .001.

Then, we focus on the roles of key transcription factors such as T-bet, GATA3, Foxp3, and STAT3, which play important roles in the differentiation of Th1, Th2, Treg, and Th17 cells, respectively. ZnCl_2_ smoke exposure decreased the expressions of T-bet and Foxp3 and increased GATA3 and STAT3 in the lung tissues, indicating the balances of Th1/Th2 and Treg/Th17 destroyed by ZnCl_2_ smoke. RES-*β*-CD prevented the imbalances of Th1/Th2 and Treg/Th17, but BUD did not (Figure 6). Therefore, RES-*β*-CD has an advantage in regulating immune response compared with BUD for the ALI induced by ZnCl_2_ smoke.

## Discussion

In this study, we successfully prepared pulmonary delivery formulation of RES-*β*-CD. The powders of RES-*β*-CD were suitable as a kind of DPIs. DPIs possess excellent properties, such as high drug loads, portability, propellant-free, and good stability (Zhang et al., 2020). However, the development of DPI formulations is a challenge for many drugs because of their high-dose needs and improper physicochemical properties. The tiny RES-*β*-CD particles became the basis of oral inhalation of the formulation with high lung deposition. The tiny RES-*β*-CD particles should result from lyophilization in RES-*β*-CD preparation. Our preliminary study showed that large RES-*β*-CD particles dominated after heat-drying at 30 °C and grinding. Therefore, freeze-drying was the optimal RES-*β*-CD preparation process. Besides the advantage of tiny RES-*β*-CD particles, the high RES entrapment efficiency and the high RES loading efficiency were shown. These properties were beneficial to pulmonary inhalation.

ZnCl_2_ smoke may lead to serious lung injury, impairment of lung liquid balance. Pulmonary edema is one of major results of lung liquid balance (Abedi et al., 2020). The external liquids of pulmonary edema may come from pulmonary vascular leakage (Slavin et al., 2018). We used an effective marker to detect the vascular leakage, i.e. Evans blue. After Evans blue is intravenously injected, it quickly binds the albumin in the circulation. If the blood vessels are injured, the protein complex of Evans blue would likely leak through the injured site (Cheng et al., 2017). The results of Evans blue and *W*/*D* ratios demonstrated RES-*β*-CD could ameliorate pulmonary vascular leakage and pulmonary edema induced by ZnCl_2_ smoke exposure.

The great release of pro-inflammatory cytokines and apoptosis were the results of ZnCl_2_ smoke exposure (El Idrissi et al., 2017). Inflammation and apoptosis further strengthen ALI/ARDS (Vadász & Sznajder, 2017), resulting in serious damage of pulmonary blood vessels, increased permeability, and a large amount of plasma proteins and active substances infiltrating to the interstitium and alveoli of the lung (Yoneshige et al., 2015). Actually, in clinical cases, the level of TNF-*α* observably increased after ZnCl_2_ smoke inhalation (Huang et al., 2008; El Idrissi et al., 2017). TNF-*α* can induce the activation of inflammatory mediators, damage granulocytes and endothelial cells, recruit inflammatory cells, and further induce tissue damage (Malaviya et al., 2017). Cigarette or wood smoke exposure would likely induce lung injury and alveolar cell apoptosis (Chen et al., 2021; Fu et al., 2021); however, ZnCl_2_ smoke-induced lung alveolar cell apoptosis had not been reported. Therefore, the inhibition of inflammation and apoptosis is important for treatment of ZnCl_2_ smoke-induced ALI. RES-*β*-CD successfully inhibited inflammation and apoptosis after pulmonary delivery due to its strong pharmacological activity and high lung deposition.

Lung tissue damage would directly weaken respiratory ability, although no specific investigations had been done on the effect of ZnCl_2_ smoke exposure on the respiratory function indices of mice. It was reported that ZnCl_2_ smoke exposure damaged the bronchi, alveoli, and blood–air barrier (Xie et al., 2019). In this study, we found that ZnCl_2_ smoke exposure reduced the lung volume, increased the airway resistance, and reduced the TV, PEF and EF50. Both RES-*β*-CD and BUD modified the levels of TV, PEF, and EF50, of which RES-*β*-CD was better than BUD. The normal physical activity of animals needs good pulmonary function and high SpO_2_. RES-*β*-CD could enhance the physical activity of the ALI mice by attenuating lung injury and increasing SpO_2_.

T cells play an important role in the immune system, especially the helper T cell subsets. CD4^+^ T cells can be categorized into four subtypes according to their own functions. Th0 cells can differentiate to Th1, Th2, Treg, and Th17 cells under different conditions. The imbalances of Th1/Th2 and Treg/Th17 are the important mechanisms in the pathogenesis of many diseases, such as ALI (Zhang et al., 2016), allergic rhinitis (Yu et al., 2017), and cancer (Lin et al., 2019). T lymphocytes subsets, especially CD4^+^ T helper cells contribute to the advancement of autoimmune and inflammatory diseases (Zhang et al., 2016). The molecular aspects involved in the pathogenesis of ZnCl_2_ smoke-induced ALI are poorly defined. This study suggested that the imbalance of Th1/Th2 and Treg/Th17 may be involved in the pathogenesis of ZnCl_2_ smoke-induced ALI. Our result is consistent with a recent study demonstrating that rats with smoke exposure showed significant imbalance in Treg/Th17 (Zhang et al., 2016). Moreover, a recent study revealed a previously unexpected role of immune responses that increase alveolar permeability in ARDS (Li et al., 2015). RES-*β*-CD has an advantage in regulating immune response compared with BUD for the ALI induced by ZnCl_2_ smoke. Besides, it must be noticed that inhaled BUD could cause immunosuppression in the localized airways (Tashkin et al., 2019).

## Conclusions

The shielding smoke produced with smoke bombs may become a major factor to impact the health of personnel in military activities and fire drills exercises; however, no appropriate treatment or prevention methods are available. Our pulmonary delivery of RES-*β*-CD shows advantages in treatment of ZnCl_2_-induced ALI from many aspects: anti-apoptosis, anti-inflammation, alleviation of pulmonary edema by healing the blood vessels, increasing SpO_2_, so that the recovery of the respiratory function is rapid and the physical activity is improved. Moreover, it regulates the balances of Th1/Th2 and Treg/Th17. Therefore, the pulmonary delivery of RES-*β*-CD is a promising strategy for prevention of ZnCl_2_ smoke-induced ALI.

## Authors contributions

Wanmei Wang, Jiahuan Li, Fei Xie, and Yiguang Jin designed the research, carried out the experiments, and performed data analysis. Yan Liu, Pan Pan, Yueqi Huang, Ting Chen, Tianyu Yuan, Yulong Ma, and Guang Han participated part of the experiments. Wanmei Wang wrote the manuscript. Jiahuan Li, Fei Xie, and Yiguang Jin revised the manuscript. All of the authors have read and approved the final manuscript.
